# Neonatal hypothermia and associated risk factors among newborns of southern Nepal

**DOI:** 10.1186/1741-7015-8-43

**Published:** 2010-07-08

**Authors:** Luke C Mullany, Joanne Katz, Subarna K Khatry, Steven C LeClerq, Gary L Darmstadt, James M Tielsch

**Affiliations:** 1 Department of International Health, Johns Hopkins Bloomberg School of Public Health, 615 N. Wolfe Street, Baltimore, MD 21205, USA; 2 Nepal Nutrition Intervention Project - Sarlahi (NNIPS), Nepal Eye Hospital Complex. PO Box 335, Tripureshwor, Kathmandu, Nepal; 3 Bill and Melinda Gates Foundation. PO Box 23350, Seattle, WA 98102, USA

## Abstract

**Background:**

Neonatal hypothermia is associated with an increased mortality risk for 28 days. There are few community-based data on specific risk factors for neonatal hypothermia. Estimates of association between neonatal hypothermia in the community and risk factors are needed to guide the design of interventions to reduce exposure.

**Methods:**

A cohort of 23,240 babies in rural southern Nepal was visited at home by field workers who measured axillary temperatures for 28 days (213,316 temperature measurements). The cumulative incidence of hypothermia (defined as < 35.0°C based on an analysis of the hypothermia-mortality risk relationship) was examined for any association with infant characteristics, care practices and parental, household, socioeconomic and demographic factors. Estimates were adjusted for age and ambient temperature.

**Results:**

Ten percent of the babies (*n *= 2342) were observed with temperatures of < 35.0°C. Adjusted prevalence ratios (Adj PR) were increased among those who weighed < 2000 g [Adj PR = 4.32 (3.73, 5.00)] or < 1500 g [Adj PR = 11.63 (8.10, 16.70)] compared to those of normal weight (> 2500 g). Risk varied inversely along the entire weight spectrum: for every 100 g decrement hypothermia risk increased by 7.4%, 13.5% and 31.3%% for babies between 3000 g and 2500 g, 2500 g and 2000 g and < 2000 g, respectively. Preterm babies (< 34 weeks), females, those who had been first breastfed after 24 h and those with hypothermic mothers were at an increased risk. In the hot season the risk disparity between smaller and larger babies increased. Hypothermia was not associated with delayed bathing, hat wearing, room warming or skin-to-skin contact: they may have been practiced reactively and thereby obscured any potential benefit.

**Conclusions:**

In addition to season in which the babies were born, weight is an important risk factor for hypothermia. Smaller babies are at higher relative risk of hypothermia during the warm period and do not receive the protective seasonal benefit apparent among larger babies. The need for year-round thermal care, early breastfeeding and maternal thermal care should be emphasized. Further work is needed to quantify the benefits of other simple neonatal thermal care practices.

## Background

Neonatal hypothermia is widely recognized as an important contributing factor to neonatal morbidity, especially in low and middle income countries [1,2] and has been associated with mortality risk in newborns [3] and young infants aged 0-2 months [4]. The World Health Organization (WHO) has included thermal care (including the prevention of neonatal hypothermia) as a component of essential care in newborn among a package of basic interventions recommended universally for all babies [5]. However, in low-resource settings, adequate thermal care of newborns is difficult to achieve and hospital-based studies in South Asia [6-9] and Sub-Saharan Africa [10-13] have demonstrated a high incidence of primary hypothermia, especially in the first 24 h after birth.

Few population-based estimates are available from settings where the majority of babies are born at home, where the risk for hypothermia is presumably higher than in hospital [9,14-16]. In southern Nepal, measurements of less than the WHO cutoff for any degree of hypothermia (36.5°C) were observed in > 90% of babies during the first 28 days of life [14]. Even less data are available on specific factors associated with an increased risk of hypothermia. The possible risks described have been ecological in nature or limited to hospital-born infants but they do indicate that preterm birth, low birth weight and seasonality (being born during the cold season) are important risk factors for neonatal hypothermia [9,10,12,16-18]. However, one comprehensive review [1] of neonatal hypothermia in low-resources settings noted a need for systematic examinations of the quantitative association between hypothermia and individual, family and community-level risk factors in large population-based cohorts.

Context-specific information on the risk factors for neonatal hypothermia is necessary in order to ensure that community-based neonatal health promotion packages include thermal care interventions for the newborn. Community-based trials of chlorhexidine cleansing interventions in southern Nepal [19,20] included repeated in-home visits by project workers to collect 213,616 axillary temperatures from more than 23,240 infants, half of whom had moderate to severe hypothermia (< 36.0°C) as defined by WHO [2,14]. In this manuscript we quantify associations between neonatal hypothermia and the specific characteristics of infants and their carers, newborn care practices and socio-demographic factors.

## Methods

### Data collection

Data for this analysis were collected during large, community-based, placebo-controlled, randomized trials of the efficacy of chlorhexidine interventions (newborn skin and umbilical cord cleansing) on neonatal mortality; the details of those trials have been previously published [19,20]. Between August 2002 and January 2006, 23,662 live-born infants in Sarlahi District, Nepal, were eligible to participate in a comparative or post-trial scale-up phase of the study. In both phases, pregnancies were identified at mid-pregnancy and oral consent was obtained. All the women received a basic set of interventions (iron/folate supplements, deworming, weekly vitamin A supplementation, clean delivery kits and basic counselling on essential newborn care). A standardized schedule of home visits (days 1-4, 6, 8, 10, 12, 14, 21, 28) was followed by the project workers. At each visit, the axillary temperature of the newborn was recorded using a low-reading digital thermometer accurate to 0.1° (Mark of Fitness, Japan Precision Instrument Co, Tokyo, Japan).

### Analytic dataset

Babies with one or more measures of axillary temperature recorded during the home visit schedule (maximum 11 visits) were included in the analysis. Data available for risk factor analysis included socio-economic variables such as caste, ethnic group (*Mahdeshi*, originating from the plains or *Pahadi*, originating from the hills), material and/or livestock ownership and parental characteristics - such as maternal birth history, parity, parental literacy, education and occupation - collected either during a population census at baseline or during the single antenatal enrollment visit. In addition, on two follow-up visits (days 1 and 14) field workers collected information on various potential determinants of hypothermia related to neonatal care practices during labour and delivery, immediately after birth and during the postnatal period. These included: birth location; type of birth assistant (family member, traditional or trained birth attendant); timing of placental delivery; sex and weight of the infant (measured to +/- 2 g with a digital scale; SECA Digital Scale Model 727); gestational age (estimated as time since last menstrual period based on maternal report at enrollment); breastfeeding initiation time [21] and thermal and skin care practices, such as washing, bathing, drying and oil massage before and after delivery of the placenta, and reported skin-to-skin contact, warming of the room and hat wearing of the infant.

Daily maximum and minimum temperature recordings were available from Simara (27°09'34''N, 84°58'47" E) and Janakpur (26°42'39" N, 85°55'27'' E) airports, which are located approximately 52 km east and 60 km west, respectively, of the geographical centre of the study area (~400 km^2^) and are at approximately 100 m altitude. The minimum ambient temperature on each day of the study period was estimated as the average minimum temperature recorded at each airport.

### Outcome variable

We have previously reported details of the burden of neonatal hypothermia in this setting as the total proportion of all axillary measures (multiple measures per child) that meet the WHO cutoffs for mild (< 36.5°C), moderate (< 36.0°C) and severe (< 32.0°C) hypothermia cutoffs throughout the neonatal period [14]. In this analysis we examine the risk factors associated with hypothermia using the same method of describing the outcome. However, the focus here is on identifying the specific risk factors associated with hypothermia and, specifically, with a degree of hypothermia clearly associated with increased mortality risk. If either of the WHO cutoffs for mild (< 36.5°C) or moderate (< 36.0°C) are used, the non-specificity of these cutoffs tends to bias estimates toward zero, not only reducing the magnitude of the association, but also obscuring potential risk relationships. Furthermore, whilst we have shown that in this setting the mortality risk is elevated after adjustments across the entire range of observed low temperature measurements, the greatest risk occurs among those with temperature of < 35.0°C [3]. Therefore, in order to improve specificity and focus on those at highest risk of subsequent mortality, we have defined the outcome for the main analyses as axillary measures of < 35.0°C.

### Analysis

All potential risk factors were separately evaluated, estimating the ratio of prevalent days across all levels of the risk factor. The large sample size enabled us to detect even small differences in the risk of hypothermia across all levels. Therefore, we required both a statistical association (*P *< 0.10) and an absolute value of the risk difference (+/- 10% risk) for the purposes of multivariate Poisson regression modelling. Final risk factor models were constructed in a three-step sequence starting with proximal infant-level factors (Model 1) and progressively adding neonatal care practices, labour and delivery characteristics (Model 2) and more distal socio-economic and parental factors (Model 3). Estimates of association in both individual factor and multivariate risk factor models were adjusted for multiple measures within infants using the Huber-White sandwich estimator [22,23]. Both individual and multivariate risk factor analyses were adjusted for ambient temperature and age at measurement as these had been pre-identified as strongly related to axillary temperature [14] and our primary interest was to identify risk factors that were potentially modifiable.

In order to further investigate the relationship between the weight of the baby and the likelihood of observing axillary temperatures of < 35.0°C, we estimated the prevalence rate ratio (PR) and 95% confidence interval (CI) for every 50-g interval between 1000 and 3450 g, compared to 3500 g. Infants who weighed less than 950 g and above 3550 g were excluded (3.4% of the sample) in order to maintain the stability of the parameter estimates. These ratios, adjusted for age and ambient temperature at measurement and gestational age at birth, were plotted to enable us to visually examine the relationship between decreasing weight and hypothermia. A final Poisson regression model with spline knots at 2000 g, 2500 g and 3000 g allowed for estimation of the increased risk of hypothermia for each incremental (1 g) decrease in weight.

In order to examine the modifying effect of the seasons on the risk factor associations, a binary indicator for birth and follow-up during the cold season (December to February) was created and the full multivariate models were first estimated separately across levels of this indicator. The full model was re-estimated by inserting interaction terms for season and any variable whose ratio of season-specific parameter estimates exceeded 20%.

All analyses were conducted using Stata 10.1 (Stata Corp, TX, USA). The Nepal Health Research Council (Kathmandu, Nepal) and the Committee on Human Research of the Johns Hopkins Bloomberg School of Public Health (Baltimore, USA) approved the protocol. The mother or other caretaker of every baby in this study gave their consent and they were informed on all aspects of the study via the use of an oral script. The parent trial is registered at http://www.Clinicaltrials.gov (NCT00109616)

## Results

Of the 23,662 live-born infants in the study area who were eligible for inclusion in the parent trial, 23,257 (98.2%) were met at one or more home visits during the neonatal period; 17 did not have any axillary measured temperatures; 23,240 contributed 213,636 axillary temperatures measures (mean 9.2 measures/infant) to the analytic dataset. There were 3134 measures of less than 35.0°C - equivalent to 1.47/100 measures (95% CI: 1.42, 1.52) from among 2342 infants, giving a cumulative risk estimate of 10.7% (95% CI: 10.3, 11.1).

All infant-level variables were strongly associated with axillary measures < 35.0°C, especially birth weight (Table 1). Compared to babies ≥ 2500 g, very low birth weight (VLBW; < 1500 g) babies were substantially more likely to be hypothermic [PR = 17.3 (95% CI: 12.6, 23.6)], as were those < 2000 g [PR = 5.34 (95% CI: 4.66, 6.12)]. Females (PR = 1.54), early preterm (< 34 weeks, PR = 4.39) and late preterm births (34-37 weeks, PR = 1.64) were also more likely to be hypothermic. Maternal and paternal education and literacy were associated with a lower prevalence of hypothermia. Hypothermia risk was not associated with either of the chlorhexidine interventions provided in the parent trial.

**Table 1:** Individual-factor associations between potential confounders and axillary temperature measures throughout the newborn period

Potential risk factor	No. < 35°C	Measures	Prevalence rate (per 100 measures)	Adjusted prevalence rate (95% confidence interval)*
**Infant-level variables**				

Sex				

Male	1271	109893	1.16	

Female	1863	103743	1.80	1.54 (1.42, 1.67)

Birth weight				

≥ 2500 g	1620	147663	1.10	

2000 - 2499 g	879	52970	1.66	1.67 (1.53, 1.82)

1500 - 2000 g	445	8854	5.03	5.34 (4.66, 6.12)

< 1500 g	160	973	16.44	17.3 (12.6, 23.6)

Gestational age				

≥ 37 wks	2281	176380	1.29	

34 - 37 wks	720	34182	2.11	1.64 (1.37, 1.83)

< 34 wks	132	2961	4.46	4.39 (3.38, 5.73)

Small for gestational age (SGA)				

Not SGA	1041	92199	1.13	

SGA	2062	118148	1.75	1.65 (1.51, 1.79)

**Parental level variables**				

Maternal age (years)				

< 15	23	996	2.31	

15.0-19.9	752	53029	1.42	0.66 (0.39, 1.09)

20.0-24.9	1169	84573	1.38	0.61 (0.37, 1.02)

25.0-29.9	686	46819	1.47	0.64 (0.38, 1.07)

30.0-34.4	348	19552	1.78	0.77 (0.46, 1.30)

35.0+	156	8667	1.8	0.77 (0.45, 1.32)

Maternal literacy				

No	2553	159538	1.60	

Yes	580	53983	1.07	0.67 (0.61, 0.75)

Maternal education (years)				

None	2589	163391	1.58	

1-3	57	4730	1.21	0.74 (0.53, 1.05)

4-6	199	15945	1.25	0.76 (0.65, 0.89)

7-9	150	13440	1.12	0.70 (0.58, 0.84)

10+	139	16130	0.86	0.58 (0.46, 0.72)

Paternal literacy				

No	1582	93307	1.70	

Yes	1551	120120	1.29	0.77 (0.71, 0.84)

Paternal education (years)				

None	1667	99134	1.68	

1-3	132	8812	1.50	0.91 (0.74, 1.11)

4-6	459	32131	1.43	0.85 (0.76, 0.96)

7-9	432	32422	1.33	0.78 (0.69, 0.89)

10+	444	41137	1.08	0.66 (0.59, 0.75)

Paternal occupation				

Farmer/unskilled laborer	2398	155637	1.54	

Skilled worker	732	57647	1.27	0.82 (0.74, 0.90)

Maternal hypothermia at baseline				

None/mild (≥ 36.0°C)	2733	193117	1.42	

Moderate/severe (< 36.0°C)	324	11128	2.91	1.45 (1.27, 1.66)

**Labour and delivery variables**				

Place of birth				

Facility	122	16405		

Non-facility	2985	189450		1.62 (1.27, 2.06)

Time to placental delivery (min)				

< 1	195	14994	1.30	

1 - 3	882	56613	1.56	1.09 (0.89, 1.32)

3 - 5	424	28785	1.47	1.08 (0.88, 1.33)

5 - 10	810	55650	1.46	1.02 (0.84, 1.24)

10 - 30	438	29472	1.49	1.05 (0.85, 1.30)

> = 30	342	17829	1.92	1.33 (1.06, 1.67)

Birth attendant				

Skilled	138	16297	0.85	

Non-skilled	2966	189232	1.57	1.47 (1.19, 1.82)

**Household and socioeconomic status variables**				

Caste				

Other castes	2822	183562	1.54	

Upper (Brahmin/Chhetri)	263	26996	0.97	0.63 (0.54, 0.74)

Ethnic group				

Hills (*Pahadi*)	626	59542	1.05	

Plains (*Madeshi*)	2454	150824	1.63	1.59 (1.44, 1.76)

Latrine				

Other/none	2828	186190	1.52	

Brick/cement	179	17820	1.00	0.71 (0.59, 0.86)

Pit latrine	73	6004	1.22	0.82 (0.62, 1.10)

Household has electricity				

No	2429	159851	1.52	

Yes	655	50540	1.30	0.87 (0.78, 0.96)

Household has television				

No	2644	172871	1.53	

Yes	440	37500	1.17	0.80 (0.71, 0.90)

**Newborn care practices**				

Breastfeeding initiation (hours)				

< 24	1452	120253	1.21	

> = 24	1595	90658	1.76	1.49 (1.37, 1.62)

Timing of cord cutting				

Before placenta	63	8219	0.77	

After placenta delivered	3018	195031	1.55	1.74 (1.31, 2.33)

Baby bathed after birth†				

No	332	21696	1.53	

Yes	2768	183386	1.51	1.18 (1.02, 1.36)

Time to giving baby first bath (hours)‡				

> 24	19	1797	1.06	

12.0 - 23.9	131	8148	1.61	0.97 (0.56, 1.71)

6.0 - 11.9	224	13407	1.67	0.98 (0.57, 1.67)

3.0 - 5.9	153	7458	2.05	1.21 (0.69, 2.12)

1.0 - 2.9	826	50679	1.63	1.05 (0.53, 1.51)

< 1	1409	101084	1.39	0.90 (0.53, 1.51)

Oil massage given after bath‡				

No	360	22407	1.61	

Yes	2397	160386	1.49	0.75 (0.66, 0.95)

Room warmed during first 14 days				

No	304	36622	0.83	

Yes	2744	175008	1.57	1.04 (0.90, 1.19)

Baby wears cap during first 14 days				

No	271	37738	0.72	

Yes	2777	173870	1.60	0.85 (0.71, 1.02)

Skin to skin contact during first 14 days				

No	2799	201332	1.39	

Yes	221	9471	2.33	1.41 (1.18, 1.68)

* All estimates are adjusted for the age of the baby and the minimum ambient temperature on the day of measurement.

† Data was collected at the time of first assessment after the birth and the timing of this visit was variable. For those met very early, respondents might report 'no bath', even though baby was probably given a bath after these data were collected.

‡ These variables only measured among those reporting a bath had been provided to the newborn.

Babies born outside a clinic or hospital (90.2% of all infants in the sample) were at a higher risk of hypothermia [PR = 1.62 (95% CI: 1.27, 2.06)], as were those born to mothers assisted at birth by non-skilled personnel [PR = 1.47 (95% CI: 1.19, 1.82)] and babies of mothers reporting at a > 30 min interval between the birth and delivery of the placenta. Upper caste, ethnic group and variables indicating improved socioeconomic status provided a greater protection from hypothermia.

Hypothermia was almost 50% more prevalent among babies for whom breastfeeding was delayed beyond 24 h [PR = 1.49 (95% CI: 1.37, 1.62)) and 75% more prevalent among babies for whom the cord was not cut until after the placenta was delivered [PR = 1.74 (95% CI: 1.31, 2.33)]. Risk was marginally higher among babies bathed after birth, an almost universal practice in this setting [24] There was little evidence that the timing of the bath within the first day of life was associated with hypothermia risk, although post-bath oil massage was slightly protective. Babies of mothers who reported warming the room, giving the infant a cap and providing skin-to-skin contact during the first 14 days were at similar risk relative to babies of mothers not reporting these practices.

In a multivariate model adjusting for neonatal-level factors (Model 1, Table 2), the association between birth weight and hypothermia attenuated only slightly, while the magnitude of the comparative risk estimates for early (< 34 weeks) and late (34-37 weeks) preterm categories were reduced on the log-linear scale by about 55%, but remained significant. Female sex remained strongly associated with hypothermia. Among delivery and newborn care practices added to the model, only delayed initiation of breastfeeding and cutting the cord after placental delivery were associated statistically with hypothermia, while all neonatal-level factors remained strongly associated. Incorporating more distal socioeconomic and parental variables (Model 3) did not change the magnitude of the associations among neonatal-level factors: females remained at 1.49 (95% CI: 1.37, 1.63) times higher risk, VLBW infants were at 11.63 (95% CI: 8.10, 16.70) times higher risk, and infants between 1500-2000 g were at a 4.32 (95% CI: 3.73, 5.00) times greater risk compared to infants weighing ≥ 2500 g. None of the socioeconomic factors was an important predictor of risk; only maternal hypothermia at first study contact after delivery was associated with higher prevalence of hypothermic measures (PR = 1.50 [95% CI: 1.32, 1.71]).

**Table 2:** Multivariate analysis of risk factors associated with axillary temperature measures < 35.0°C among newborns of Sarlahi

Factor	Model 1 (*n *= 210,347)	Model 2 (*n *= 196,045)	Model 3 (*n *= 190,180)
**Neonatal-level**			

Female (versus male)	**1.47 (1.36, 1.60)**	**1.48 (1.36, 1.61)**	**1.49 (1.37, 1.63)**

< 1500 g (versus ≥ 2500 g)	**14.50 (10.60, 19.84)**	**12.60 (8.85, 17.93)**	**11.63 (8.10, 16.70)**

1500 - 1999 g (versus ≥ 2500 g)	**4.68 (4.09, 5.35)**	**4.38 (3.80, 5.05)**	**4.32 (3.73, 5.00)**

2000 - 2499 g (versus ≥ 2500 g)	**1.57 (1.44, 1.72)**	**1.51 (1.38, 1.65)**	**1.49 (1.36, 1.63)**

34 - 36.9 weeks (versus ≥ 37 weeks)	**1.24 (1.12, 1.38)**	**1.19 (1.07, 1.32)**	**1.16 (1.04, 1.29)**

< 34 weeks (versus ≥ 37 weeks)	**1.94 (1.53, 2.46)**	**1.85 (1.42, 2.40)**	**1.87 (1.44, 2.44)**

			

**Delivery/newborn care practices**			

Non-facility birth		1.35 (0.93, 1.96)	1.28 (0.88, 1.88)

Non-skilled attendant		0.97 (0.69, 1.35)	0.97 (0.68, 1.37)

Delayed (> 24 h) breastfeeding		**1.25 (1.15, 1.35)**	**1.19 (1.08, 1.30)**

Cord cut after placenta delivered		1.45 (0.97, 2.18)	1.47 (0.98, 2.22)

Baby bathed after birth		1.03 (0.88, 1.20)	1.02 (0.87, 1.20)

Room warmed during first 14 days		0.97 (0.85, 1.11)	0.96 (0.84, 1.11)

Hat worn during first 14 days		1.08 (0.91, 1.28)	1.10 (0.92, 1.30)

Skin-to-skin during first 14 days		1.11 (0.92, 1.33)	1.10 (0.92, 1.31)

			

**Household/socioeconomic status variables**			

Upper caste (versus lower)			0.88 (0.73, 1.06)

Madeshi (versus Pahadi)			1.04 (0.91, 1.19)

Latrine (brick/pit versus no latrine)			1.05 (0.92, 1.19)

Electricity (versus none)			1.07 (0.95, 1.21)

Television (versus none)			0.94 (0.82, 1.09)

			

**Parental variables**			

Maternal literacy (versus none)			0.90 (0.79, 1.02)

Paternal literacy (versus none)			0.99 (0.90, 1.09)

Paternal occupation (skilled versus non- skilled)			0.97 (0.87, 1.07)

Maternal hypothermia (< 36.0°C)			**1.50 (1.32, 1.71)**

### Hypothermia risk associated with incremental weight differences

**Figure 1:**
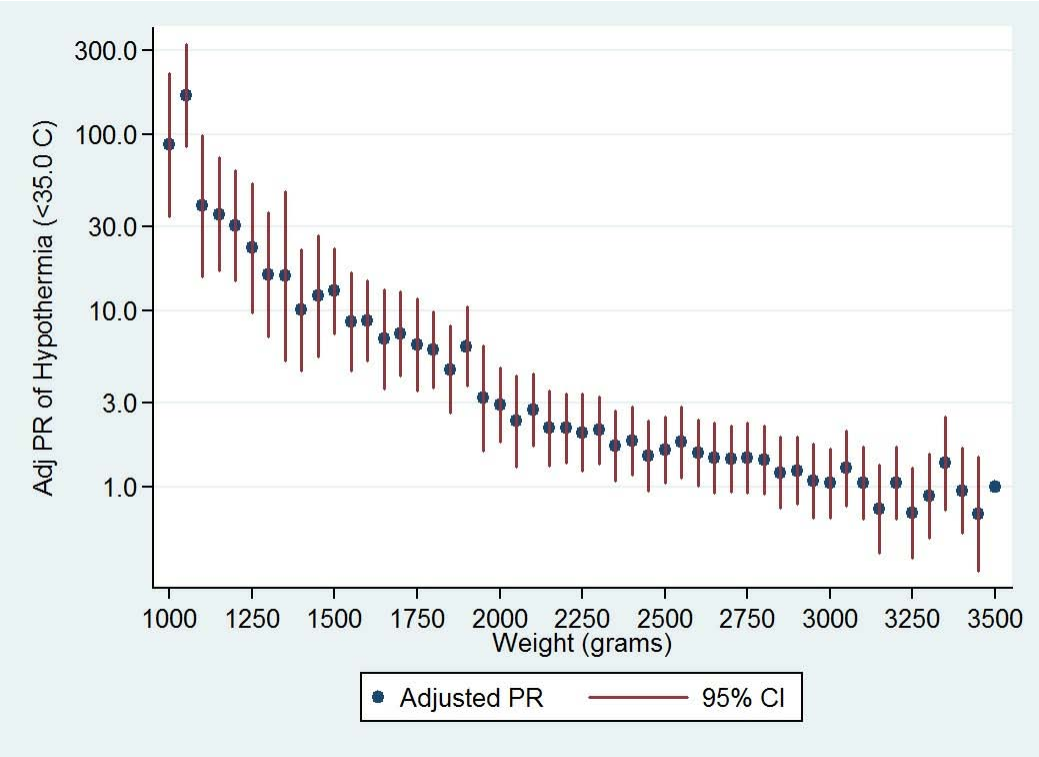
**Adjusted prevalence ratios for axillary temperature < 35.0°C, by weight interval**. NB: Ratio adjusted for age at measurement, ambient temperature at measurement and gestational age at birth; reference weight 3500 g.

Given the strength of the association with infant weight, the risk associated with incremental (50 g) changes in weight was examined (Figure 1). After modelling the risk associated with weight as a continuous measure in a spline Poisson regression model, there was only weak evidence that weight of between 3500-3000 g was associated with hypothermia risk (*P *= 0.09). However, each 100 g decrement in weight between 3000 g and 2500 g was associated with a 7.4% (95% CI: 4.4%, 10.5%) increase in risk of hypothermia and the association became substantially stronger and of greater magnitude between 2500 g and 2000 g [13.5% (9.7%, 17.5%)] and < 2000 g [31.3% (26.3%, 36.5%)].

### Influence of seasonality on risk factor analysis

The season during which a child was born was examined for interaction terms for birth weight category, delivery location, birth attendance with skilled personnel, hat-wearing during the first 14 days and caste and ethnicity. There was evidence that the season during which babes were born modified the association between hypothermia and the two lowest birth weight categories and hat wearing by the infant (interaction *P*-values: < 0.001, 0.06 and 0.003, respectively). In the warm season, relative to newborns ≥ 2500 g, the adjusted prevalence of hypothermia was 23.46 (95% CI: 16.49, 33.37) and 5.05 (95% CI: 4.14, 6.16) times greater in the lowest (< 1500 g) and second-lowest (2000 g-2499 g) weight categories, respectively. The parallel ratios of adjusted prevalence (Adj PR) during the cold season were less: 4.04 (95% CI: 2.34, 7.00) and 3.80 (95% CI: 3.07, 4.71), respectively. Similarly, wearing a hat was protective only during the cold season [Adj PR = 0.71 (0.52 - 0.96)].

## Discussion

In a low resource setting in southern Nepal, where 90% or more of births occur at home, after adjusting for the age at measurement and ambient temperature (the season), the weight of the infant was the most important independent factor related to neonatal hypothermia. Almost 30% of infants had low birth weight (< 2500 g) and those between 2000 g -2499 g and 1500 g -1999 g were at a 1.49 and 4.32 times greater risk of hypothermia, respectively, after adjusting for a multitude of factors. VLBW babies were 11.63 times more likely than normal weight infants to have axillary temperatures < 35.0°C, although the proportion of these babies was very low (146 of 22,748, 0.6%). When examined as a continuous variable, 100 g decrements in weight were associated with substantial increases in risk of hypothermia along the entire spectrum of underweight babies and continued up to 3000 g. Such associations have been documented previously among hospital-born newborns [12,25,26].

All newborn babies are prone to heat loss, particularly in the first minutes and hours after birth [1,2]. Preterm and underweight babies lose heat faster than normal weight or full term babies, due to larger surface to mass ratios [1] and rapid transepidermal loss of water through the compromised skin barrier [27]. Preterm birth and low weight were independently found to be related to hypothermia in these data but the latter appeared to a more important factor; in both cases these infants are more susceptible to hypothermia due to their reduced brown adipose tissue reserves compared to full terms infants with appropriate weight for gestational age. Given that [1] much underweight at birth is due to intra-uterine growth restriction in this setting, [2] the 'normalcy' of small babies in this community relative to early birth (especially very early preterm infants < 34 weeks) and [3] the potential differential capacity to recognize early birth versus small size at birth [28], the intention and timing (and, thus, potential causality) of thermal care practices such oil massage, warming the room and provision of a hat may differ for these babies. Our data on these specific practices are limited by uncertainty as to whether the practice resulted as a therapeutic (reactive) response to the caretaker's perception of her baby being cold or as a preventative measure. Differential intent (more therapeutic versus prophylactic) of these and potentially other non-measured practices between preterm and low birth weight babies might contribute to the observed greater importance of weight as a risk factor.

These weight measures (precise to +/- 2 g) are more objective than gestational age, which was estimated from the date of the last menstrual period reported by mothers at enrollment at approximately 6 months. Thus, there is more scope for misclassification of gestational age, which could result in estimates of the association with hypothermia that are biased downwards [29]. While weight varies over the newborn period, 89.6% of weight measures were recorded within 72 h of birth and hypothermia risk factor models were adjusted for age at every measurement. Limiting the dataset to only infants measured within 72 hours of birth did not substantially change any risk factor estimates or conclusions.

Adj PRs for the lowest birth weight category were five times higher in the warm season than in the cold. This indicates that, while the overall risk of hypothermia is lower during this time relative to the cold months, [14] low birth weight babies are disproportionately at risk and do not receive the protective benefit of the warmer months. Cold season practices, such as warming the room or providing baby with a hat, did not appear to be protective in this setting possibly due to reverse causality (that is, that these are more likely practiced as reactive measure to the caretaker's perception that the baby is cold). However, although caretakers are generally more aware of the importance of keeping the baby warm during the cold months, the absence of such attention during warm periods may differentially impact low birth weight infants, resulting in the substantially increased magnitude of comparative risk estimates among these babies during the warm periods.

The strong association between hypothermia and the sex of the newborn is not easily explained. There may be unmeasured practices that differ according to the sex of the newborn which may change the risk profile [30]. Alternatively, if observed care practices (such as skin-to-skin contact and hat wearing which did not differ by sex) are conducted with *differing intent *between boys and girls (as being preventive for boys but therapeutic for girls), these sex-related associations could arise even after adjustment.

Delayed breastfeeding was associated with hypothermia after adjustment: early breastfeeding may directly reduce risk through this additional direct contact. In addition, early breastfeeding has been associated with reduced neonatal mortality [21,31] potentially through a reduced risk of infection and hypothermia.

While kangaroo-mother-care or skin-to-skin contact is recommended as part of a package of targeted care for low birth weight or preterm infants in facilities [32] debate continues as to the benefit of extending this to all infants in community settings [9,33]. Maternal report of skin-to-skin contact during the first 14 days after birth was not related to the risk of hypothermia. However, this practice is not part of usual care in this community (only 4.4% of infants received this care) and may be a reactive practice undertaken when mothers perceive their baby to be cold. Those receiving this care were more likely to have initiated breastfeeding after 24 h, providing some support for the reactive nature of the practice.

If adequate thermal protection is not begun immediately after birth, the newborn will lose heat rapidly, with the skin temperature losing up to 4°C in the first minutes [34]. Thus, immediate actions, such as wrapping and drying the baby without waiting for the placenta to be delivered, are recommended as part of essential newborn care [2,5]. In our setting, newborns are often left unattended until after the placenta is delivered. Both delayed placental delivery and the fact that the cutting of the cord occurs after placental delivery were associated with an increased risk of hypothermia. However, as delayed cutting of the cord may improve iron status and reduce the risk of anaemia, [35] early cutting should not be encouraged. Rather, further emphasis should be placed on encouraging attendants to rapidly dry and wrap the newborn, to have skin-to-skin contact with the mother and to avoid leaving the baby unattended during the period between birth and delivery of the placenta.

## Conclusions

These data provide an in-depth examination of the risk factors associated with neonatal hypothermia as measured by axillary temperatures collected at home from 23,240 infants. The data highlight the importance of thermal care interventions, especially those targeted towards low birth weight infants. Focused behaviour change messages and skill building on the basic components of thermal care, including early breastfeeding initiation, skin-to-skin contact for all infants and delayed bathing have been promoted in Shivgarh, India and have been resulted in a 54% reduction in neonatal mortality [36]. The relevance and specific importance of these interventions for underweight, preterm and other high risk infants need to be emphasized. Further evaluations of the delivery mechanisms of behavioural change communications which stress the importance of thermal care and skin-to-skin contact within the community is necessary. Estimates of the risk of adverse outcomes such as mortality and morbidity of early exposure to neonatal hypothermia in the community are urgently needed.

## Abbreviations

Adj PR: adjusted PR; CI: confidence interval; LBW: low birth weight; PR: prevalence ratio; VLBW: very low birth weight; WHO: World Health Organization.

## Competing interests

The authors declare that they have no competing interests.

## Authors' contributions

LCM contributed to the concept, design, conduct and analysis of the study and also wrote the manuscript. JK contributed to the overall study concept, design, interpretation and provided a critical review of the manuscript. SKK and SCL contributed to the design and implementation of the study, provided an oversight of data collection and critically reviewed the manuscript. GLD contributed to the overall study concept, interpretation and critical review of the manuscript. JMT provided inputs on the overall concept and design of the study, the interpretation of the results and a critical review of the manuscript. All authors read and approved the final manuscript.

## Pre-publication history

The pre-publication history for this paper can be accessed here:

http://www.biomedcentral.com/1741-7015/8/43/prepub
